# The Health Literacy of First Year Physiotherapy and Speech Pathology Students and Their Perceived Future Roles in Supporting Their Clients’ Health Literacy

**DOI:** 10.3390/ijerph20116013

**Published:** 2023-05-31

**Authors:** Romany Martin, Jade Cartwright, Marie-Louise Bird

**Affiliations:** School of Health Sciences, University of Tasmania, Launceston, TAS 7250, Australia; jade.cartwright@utas.edu.au (J.C.); marie-louise.bird@utas.edu.au (M.-L.B.)

**Keywords:** health literacy, allied health, health promotion, physiotherapy, speech pathology, Australia

## Abstract

Background: Allied health professionals are well positioned to assess and support their clients’ health literacy (HL); however, they report being deficient in HL knowledge and skills. Objective: To explore allied health students’ HL and their perceptions of their roles in supporting clients’ HL. Design: A mixed-methods cross-sectional study was undertaken in August 2022 amongst allied health students enrolled in graduate-entry masters programs at the University of Tasmania. Data collected included a Health Literacy Questionnaire (HLQ) (*n* = 30) and qualitative telephone interviews (*n* = 6). Results: Allied health students’ confidence in the knowledge domain of the HLQ was rated as 28.57 from a maximum possible score of 50. Similarly, the students’ confidence in the skills domain of the HLQ was rated as 14.87 from a maximum possible score of 25. Four themes were generated from the qualitative interviews: (1) valuing HL, (2) an innate part of their future roles, (3) contributors to their own HL, and (4) advocacy and their decision to study allied health. Conclusion: This study provides a preliminary insight into the HL of allied health students and highlights the strong perception held by allied health students that supporting clients’ HL is a large component of their future roles.

## 1. Introduction

Health literacy (HL) refers to the ability to access, understand, and apply information and services to make decisions about healthcare [1]. In recent decades, HL has become the focus of multiple research efforts aiming to improve how healthcare is designed and accessed, secondary to the significant impact that HL has on health outcomes [2]. A systematic review by Berkman et al. demonstrated that low HL is associated with increased hospitalizations, greater use of emergency care, and in geriatric populations, poorer overall health status and higher mortality rates [3].

Allied health professionals (AHPs) represent approximately 20% of Australia’s healthcare workforce and play important roles in improving client outcomes and minimising harm from injury [4]. The term AHP is commonly understood to include all healthcare disciplines outside of medicine and nursing, and can be either therapy focussed, such as physiotherapy and speech pathology, or investigation focused, such as medical laboratory science and radiology. A 2009 literature review by Hester and Stevens-Ratchford examined the role of speech pathologists in HL, highlighting that limited research has investigated HL in people with communication disorders and their families. They proposed a conceptual framework to support speech pathologists’ involvement in HL and emphasised the profession’s role in preparing intervention materials to improve HL in people with and without communication disorders [5]. Similarly, physiotherapists are encouraged to embrace their roles in supporting clients’ HL and be responsive to HL during client education through avoiding medical jargon and using the teach-back cycle [6]. Ensuring that AHPs are prepared and supported to appropriately respond to the HL of their clients may help to overcome the barriers that are faced by clients with low HL [7].

Despite the acknowledgment of the value in HL screening and supporting clients’ HL [3], healthcare professionals continue to inadequately respond to or prioritise the HL needs of their clients [8]. Research suggests that contributing to this lack of health literacy responsiveness is not only a lack of HL content in pre-professional training [9], but also significant gaps in health professionals’ own HL [10]. A study of American speech pathology and audiology clinicians and students reported that they were aware that low HL is an obstacle for clients; however, they were unaware of the prevalence amongst their client population and were unaware of communication approaches to address low HL, for example, the readability of clinic forms [11]. A 2018 systematic review by Rajah et al. reported that out of 17 studies, 13 reported that health care professionals had deficiencies in HL-related knowledge [8]. Rajah et al. (2018) concluded that immediate intervention is required, for example, through providing opportunities for healthcare professionals to learn about HL [8] so that they could address health literacy in their usual approaches to health promotion.

The development of health literacy skills occurs on a continuum throughout training and in the post-graduate context. This has important implications for tailoring health professional curricula in the pursuit of HL-based competence, as students enrolled in the post-graduate entry-level master’s programs have higher HL scores than those enrolled in bachelor’s programs [12]. Similar qualitative cross-sectional studies support the notion that HL is developed following explicit training within health professional education [13]. While a set of competencies for health literacy curricula have been developed and used frequently in medical and nursing training, the existing HL knowledge and skills, and hence training needs, for allied health students at the commencement of their programs appears less often in the literature [14]. Understanding the HL of allied health students at the commencement of their studies may inform development of curricula and teaching and learning experiences with potential to positively shape HL perceptions and practices.

Despite the benefits of being responsive to HL [2] and the need to prepare AHPs to work in this space [11,15], to the authors’ best knowledge, the HL of allied health students and their perceptions of their roles in supporting clients’ HL are not known. The need for this research is further supported by a 2019 systematic review which concluded that HL is an underdeveloped domain in health professional education and that further research is required to determine the optimal time period for developing HL-related competency during pre-professional training that may then enable targeted HL curricula [9]. Understanding allied health students’ perceptions of HL at the commencement of their pre-professional training may inform the content and pedagogical design of HL education provided. Therefore, this study aimed to explore:

(1) first year allied health students’ HL,
(2) their perceptions of their roles in supporting their clients’ HL, and
(3) any relationship between their own HL and their decision to study allied health.

## 2. Materials and Methods

A convergent parallel mixed-methods design was undertaken to achieve the aims of the study, with pragmatism as an umbrella philosophy [16]. A health literacy questionnaire (HLQ) [15] which has been previously used to assess HL knowledge and skill amongst practicing physiotherapists was administered via hard-copy survey. The survey was originally created by the Centre for Culture, Ethnicity, and Health [17]. Additionally, qualitative telephone interviews were undertaken and followed a semi-structured interview guide. The sample studied was post-graduate students enrolled in either a graduate-entry master of physiotherapy program or a graduate-entry master of speech pathology program at a regional Australian university. Ethical approval was provided by the University of Tasmania Human Research Ethics Committee—Approval Number #27665.

### 2.1. Participants

Participants were purposefully recruited and were required to be newly enrolled in either a graduate-entry master of physiotherapy or a graduate entry master of speech pathology at the University of Tasmania. Both programs were in their inaugural years of enrolment and are part of the Allied Expansion Program at the University of Tasmania, which aims to increase the capacity of the University to educate AHPs such as occupational therapists, nutritionists, and dieticians. There were no other inclusion criteria to be involved in the study. Students in their first month of enrolment were chosen as potential participants, as the research aimed to explore the health literacy of students prior to their immersion in health professional education. This allowed the research to achieve the third aim of the study, being the exploration of potential relationships between HL and the decision to study allied health. Participants were approached regarding the research during an in-person practical class by a staff member not involved with the teaching of the practical class nor the unit of study that the practical class was associated with. The staff member (RM) was known to some students. Participants were informed that their decision regarding participation would not influence their current or future relationship with the university in any way.

### 2.2. Data Collection

The HLQ chosen has featured in the literature and has been used to identify the HL skills and professional development needs of physiotherapists [15]. Whilst the HLQ used in this study aims to measure the HL of survey respondents similar to other HLQ tools [18], it may also measure elements of health literacy responsiveness. The HLQ was chosen for this cohort study, given the similar aim of identifying the HL education needs of allied health students. As part of the approved ethics application, the research team plans to include the HLQ data featured in this study in a future study that evaluates the influence of a two-year health literacy curriculum on students’ longitudinal health literacy.

A semi-structured interview guide (Table 1) was designed by the research team, following a review of the extant literature. The research team has expertise in HL, pedagogical design, and teaching and learning research amongst allied health students. The interview guide was designed to capture a range of both positive and negative perspectives from the participants and was piloted within the research team. All participants were provided with a definition of HL at the beginning of the interview, taken from Dodson et al. [1]. This definition can be seen below:

*HL is described as; the personal characteristics and social resources needed for individuals and communities to access, understand, appraise, and use information and services to make decisions about health. Health literacy includes the capacity to communicate, assert and enact these decisions (pg. 12)*.

[1]

If survey participants were interested in participating in a telephone interview, they were invited to provide their email address on a separate piece of paper that was able to be deattached from the survey responses to ensure that they remained anonymous. Potential interivew participants were then emailed by the lead researcher (RM) with additional information regarding the interview. Written consent was obtained from all interview participants, with interviews scheduled at a mutually convenient time. All interviews were conducted by the lead researcher (RM) via telephone and were audio-recorded on a secondary device to allow for accurate transcription. Information power was, in part, determined by the researchers’ interpretation of the data and was also determined by the availability of consenting participants. Malterud et al. describe five principles to guide appropriate power for a qualitative study: the breadth of the aims of the study, the sparsity of the potential participant group, established theories that support the research, the quality of the dialogue, and the analysis strategy undertaken [19]. With consideration for the specific aims of the study, the quality of the diagloge, and the specificity of the participant sample of interest, the researchers felt that information power was achieved for the results that are presented [19].

**Table 1:** Example Interview Guide.

Example Interview Guide:
Can you comment on your own level of health literacy? Do you feel positive or negative about your own level of health literacy? What do you think has contributed to your own health literacy? Why do you think this? Do you think healthcare professionals have a role in health literacy? Do you think that allied healthcare professionals have a role in health literacy? Do you think that your health literacy informed your decision to study allied health? If so, why, or why not? How did your health literacy inform your choice? *Probing Questions*: Can you elaborate? Why do you think that might be? Are you able to provide an example?

### 2.3. Data Analysis

Survey data from the HLQ were inputted into Microsoft Excel, from the hard-copy surveys on which they were collected. Microsoft Excel was then used to calculate the mean and standard deviation. Data were rounded to 2 decimal points as the precedent set in the literature [15].

Interview data were subject to reflexive thematic analysis, with the aim of generating themes that communicate patterns of shared meaning [20]. Prior to immersing themselves in the data, both researchers undertook the process of epoch whereby they identified their prior knowledge, beliefs, and experiences relevant to the phenomenon on interest [21]. This process of epoch allowed the researchers to reflect on how their own perspectives and lived experiences influenced their interpretation of the data. The lead researcher (RM) is a current teaching and research academic with a doctorate, who has practiced as both an inpatient and outpatient physiotherapist and has four years of experience in qualitative research. The other researcher undertaking the analysis (JC) is a current teaching and research academic with a doctorate, who has expert knowledge in speech pathology teaching and learning and care for people with dementia. Both researchers have previously published research relevant to teaching and learning. Following the reflexive approach detailed by Braun and Clarke (2021), six repetitive phases were undertaken. Initially, the researchers immersed themselves in the data during the transcription process through reading the complete transcripts of the interview to familiarise themselves with the data. On subsequent readings of the data, the researcher annotated the transcripts through identifying recurring ideas and concepts in the form of codes. Initial themes were then generated via grouping codes that summarised similar concepts discussed by the participants. The full research team met to review and further develop the themes on two separate occasions to ensure that the results were grounded in the data. The themes were refined until consensus was reached amongst the research team, and the themes were subsequently named [20].

Multiple processes were undertaken to ensure the validity of the qualitative analysis. All interviews were conducted with reference to the interview guide and were audio-recorded, and all data were transcribed verbatim. Participants were provided with a written transcript of their interview data via email and were invited to review the transcript to ensure that it was reflective of their views. No changes were made following this process. Other processes undertaken to ensure the trustworthiness of the analysis process include independent analysis by two researchers and the discussion amongst the research team until consensus was reached regarding the codes and themes.

## 3. Results

All 32 potential participants who were approached regarding the survey consented to participate in the HLQ. The sample consistent of 22 students enrolled in the graduate-entry master of physiotherapy and 10 students enrolled in the graduate-entry master of speech pathology. The data collected from the 32 students represented 97% (32/33) of the allied health students at the university. At the time of data collection, there were 33 students enrolled across both programs, and on the day of data collection, one student was not in attendance. Both programs were in their inaugural year of operation, and the university is geographically located in a regional centre. Further demographic information is available in Table 2. Of the 32 surveys that were returned to the researchers, 2 were incomplete and were subsequently removed from the dataset. Due to the demographic information being collected separately and the autonomous nature of the survey, these two participants were unable to be removed from the data regarding the demographic characteristics of the sample. The results of the HLQ are presented in Table 3. The knowledge and skills domains of the HLQ are presented in Table 4.

**Figure 1:**
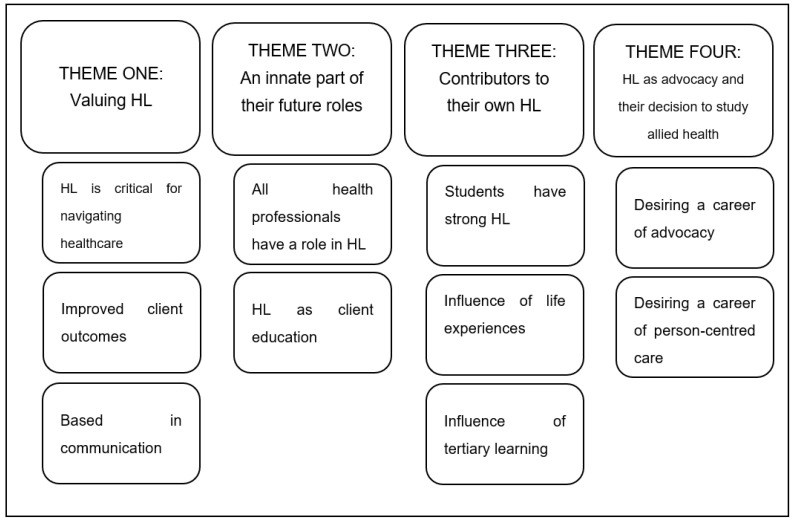
Themes and sub-themes related to perspectives of health literacy.

A total of 11 students provided consent to be contacted regarding a telephone interview, 8 of which responded to the initial email sent by the lead researcher. Two students withdrew from the interview, citing time constraints. A total of six participants consented to be interviewed via telephone, and the demographic details of each participant are available in Table 5. The interviews ranged from 14 min to 20 min with a mean interview time of 18 min. Four themes were identified that together represent allied health students’ perceptions of HL and their roles in supporting clients’ HL. These themes and their corresponding codes are detailed in Figure 1.

### 3.1. Theme One: Valuing Health Literacy

All participants saw the value of being health literate, as HL was perceived to be a “*critical*” (P2) component of successfully navigating health care and promoting “*good health and wellbeing”* (P2). Participants discussed health communication as being central to HL and emphasised the importance of adapting communication to the needs of each individual client. For example, P4 voiced that “*Having always access to it* (health care) *is one thing, but you can access it and still not understand it*”.

*I’ve sort of understood how important communication is for each individual client and that some clients require some a different level of health literacy, to be able to understand*.

(P6)

*I suppose you need to be health literate in order to understand … to interpret information about your health, say, from a health professional, and to know, sort of what to do with that information. I think that that’s really critical because if you don’t have an understanding or if health professionals use jargon … it’s really important, on a personal level, so that people understand … what is good for health*.

(P2)

Extending the idea of tailored communication, participants identified that every person has a “*different level of HL”* (P6) and that a responsive and person-centred approach to HL helps to improve health care outcomes and engagement in shared and informed decision-making.

*I definitely do think it does (have an impact on health outcomes) but I think you have to treat clients as an individual … so being able to sort of tailor your communication style to that person will impact on the health outcomes*.

(P6)

*Health literacy is important for understanding health outcomes and health language and all that sort of thing, so it makes sense that health literacy is a big part of it, because it helps us make more informed and better choices*.

(P1)

Furthermore, participants perceived that health care that was responsive to the HL of a client was more respectful and “*a lot less overwhelming for the client*” (P6), associated with improved satisfaction with health services, greater trust, and stronger therapeutic relationships.

*If they’re already concerned, apprehensive about coming in and worried about, you know, what’s wrong with them, that if you can provide them an understanding that’s to their level of health literacy … they’re going to trust you a lot more and then be a lot less apprehensive during treatments. And I think it’s just positive for the client and the therapist, for the interaction in that building relationship, that you’ve taken the time to sort of make sure that can understand, you know, what your role is and what, what they’re trying to do to help you and how they can help you*.

(P6)

### 3.2. Theme Two: An Innate Part of Their Future Roles

Participants situated HL as a “*part of everyday practice as a health professional”* and voiced that it “*makes sense*” (P1) that AHPs had a role in HL screening and supporting clients’ HL in the pursuit of optimal client outcomes. Contributing towards HL screening and supporting clients’ HL was repeatedly reinforced as a role that all health professionals should adopt.

*It makes sense that they should (contribute towards health literacy) … if we’re all sort of contributing to health literacy … then we can make those better choices and people can make more informed decisions about their own health and the future of health … So it does make sense that if everybody is contributing to that, then we can build something together*.

(P1)

*Yeah, I think absolutely. I think that it (improving health literacy) is a part of any health profession really or it should be. Because … you can help people, but you also need to ensure, as I’ve said that they’re understanding the message, and that they go away feeling satisfied with the experience … I think it’s a part of everyday practice as a health professional*.

(P2)

The participants reflected that health care professionals must be responsive to a client’s HL to enable core processes, such as informed consent, because without adapting their communication to the needs of the client, informed consent was not achieved. Further, participants drew links between improving a person’s HL and promoting the self-management of health more broadly. This was discussed in the context of the participants’ belief that healthcare was moving away from traditional power structures, towards client advocacy and “proactive” (P4) health care.

*All healthcare workers do (have a role in health literacy) … it’s part of informed consent, making sure that the client understands everything that’s happening to them, all the treatments that they’re agreeing to, that sort of thing*.

(P3)

*Ultimately, if people can take care of their own health, and that’s the ultimate sort of role of allied health professionals is to get people self-sufficient, and rather than continuing to come back … I guess that’s the goal, really … to increase people’s understanding so they can take care of their own health*.

(P5)

Participants identified skills and tools that support their role in supporting clients’ HL and providing accessible communication, including the teach-back approach. They also acknowledged their role in providing information regarding other available health services and professionals as a way of supporting HL.

*Obviously talking to them and explaining and getting that sort of teach back thing to see if they understand … and then giving them good sources, information, reliable sources, and referring them on to people in other professions and knowledgeable*.

(P3)

*They know what to expect. They know what other health professionals they can contact and what their referrals mean, and things like that*.

(P2)

Whilst having a role in supporting HL was not anticipated, the participants viewed HL as analogous to their anticipated roles in client advocacy and client education. One participant reflected on an experience seeing a physiotherapist as a client and stated that they were “*frustrated about not being educated*” (P5), which motivated him to include HL-focused client education in his future work as a clinician.

*I think that it (heath literacy) is something that I’m aware of how important it is to be able to develop that communication and provide education to the client, so it’s something that I was looking forward to developing, becoming a physio*.

(P6)

*For instance, the further down the northwest coast, you go, the worse the health literacy is. And I think yeah, physiotherapy has a huge part to play in education and supporting peoples’ self-efficacy*.

(P4)

On a broader level, participants also voiced that their roles in HL were grounded in an interprofessional framework whereby all members of the team working with a person have a role in contributing to supporting clients’ HL as part of their roles in health promotion. Furthermore, participants emphasised the importance of all team members communicating the same message, which relies on effective communication within the inter-professional team.

*Sort of finding a common framework through which, you know, speechies and physios can think about the client to then pass on information to the client and ensure that they understand what’s going on. And then it’s not just coming from two different completely different perspectives and sort of languages*.

(P2)

### 3.3. Theme Three: Contributors to Their Own Health Literacy

Participants felt “*positive*” (P3) about their own HL and felt that they were able to navigate the health care system with relative ease.

*I feel like I’ve come from a position of privilege, you know, I’m educated, and I’ve had access to really, really good accessible health information*.

(P2)

*I just understand what I have access to, privately, publicly, waiting times, where I need to get information. It’s just yeah, very easy for me*.

(P4)

Whilst reflecting on their HL, participants identified multiple factors that had contributed to their development of HL, including the social and cultural variables of their upbringing and the education that they had undertaken to date.

*Probably to begin, with my family … They have a very sort of scientific and pragmatic background, and they were very up to date with my health care … ever since I was a baby I’ve been vaccinated, that sort of thing. And then schooling, obviously, university, my job … It’s just the social environment that I’m in (that dictates) what I’ve learnt about it (health literacy)*.

(P3)

*I feel like my health literacy is quite high … suppose I was always, raised quiet, you know, middle class, and I was always sporty, so I always had access to like, doctors, physios … and then obviously, going to uni and then improving my own health literacy there*.

(P4)

Participants strongly felt that their health-specific units of study at university contributed to their own HL, their ability to be “*critical of health information”* (P2), and their understanding of HL in their roles as health professionals. The notion that HL was discipline specific tied back to the importance of an interprofessional approach to supporting clients’ HL.

*I have sort of a background in more like psychology and that sort of stuff, so my health literacy sort of revolves around that aspect*.

(P1)

*I think the education component is huge, like, undergrad, we had a heap of health literacy, health promotion units. And I think I probably didn’t appreciate at the time when I was completing them how important that they are*.

(P4)

*I think health literacy is very field specific, so I’d have no idea about anything than anaesthesiologists would do or anything like that*.

(P3)

One participant felt that their age was a major contributor to their HL, which was attributed to their life experience in having to navigate the health care system as an adult.

*I definitely think it’s skewed by age. I think the older like, some of the older students have a much more better understanding of what’s accessible. And how to access it … For like me and even a couple of late 20s students, have a much better understanding of like, living out of home and having to access things for yourself. And whereas I think some of the younger ones, potentially not, but I still think they’re above general population of health literacy*.

(P4)

Despite feeling positive about their HL, participants acknowledge that their HL can always improve and there is no ceiling effect for HL learning.

*I don’t think my health literacy is amazing. I don’t think it’s the top, but I think, yeah, I do feel very privileged and lucky that I have had access to education, because that’s what that’s how we become health literate*.

(P2)

*But, you know, there’s so many different domains of health that, you know, my health literacy could definitely expand and improve*.

(P1)

Understanding HL as an evolving and ever-improving metric was a concept that participants also applied to their roles as health care professionals, as they felt that the HL of clients could also continually improve.

### 3.4. Theme Four: HL as Advocacy and the Decision to Study Allied Health

Participants felt that their own level of HL had not directly influenced their decision to enrol in an allied health course; however, they voiced that their understanding of the role of AHPs in advocacy was a factor that influenced their decision to study allied health. This was attributed to their desire for a career that allowed them to “*help*” (P1, P2, P4) people.

*I’ve always wanted to help people. I think that was sort of like, if I were to think of a job, that would be the most fulfilling and would give me a sense of purpose, it would definitely be something that helps people. It was just a matter of choosing which direction to go in, I suppose. I just found that speech pathology seems like a really like, positive, progressive kind of profession, you know, like, you’re really helping someone get from one point to a point where you really like improving their quality of life … giving them a better future or helping them maintain, you know, something positive*.

(P1)

*But I’m always looking for the ways to improve my skill set so I can then offer them (clients) more, so I suppose that’s one of the reasons probably the biggest reason why I did sign up for physio*.

(P4)

Furthermore, there was a perception that AHPs focused on person-centred care, which also contributed to the participants’ decision to study allied health.

*I knew it wasn’t just, you know, perform this test, and, you know, assess this, this area of the body, it’s more so being able to engage with the client. And, you know, that’s, I think that’s important attribute to have. Yeah, yes, it sort of did draw me to physio*.

(P6)

*I definitely didn’t sign up for physio, just to do like, solely manual therapy, diagnostic, acute exercise stuff. It’s more about being with patients, on the whole journey, as opposed to just halfway coming through*.

(P4)

Factors that contributed to their perception of allied health as a career that included advocacy and altruism included experiences in their prior workplaces, exposure to AHPs through social contacts, and explicit research regarding the role of allied health.

*I am a disability support worker and my client was going to speech pathology sessions, and I … was just inspired by the work that her speech pathologist could do with her. And I really want to do that. I want to make that change …. I have been really, really inspired by especially in, in our speech pathology specific classes, We’ve learnt a lot about advocacy and working with communities. That’s been massive. And that, I guess that has inspired me more and more throughout the course as well as prior to beginning the cause*.

(P2)

*I’ve come across like other allied health professionals like friends and family that are allied health professionals, and I did a lot of research into the role as well, I sort of come across it while I was thinking about becoming a psychologist, actually … it just seemed very positive. And like, they did a lot of good work to help people get better*.

(P1)

## 4. Discussion

This study has explored the HL of allied health students and investigated their perceptions of their roles in supporting clients’ HL. When reflecting on their perceptions of HL, allied health students voiced that HL was a fundamental underpinning of health and felt that their own HL was influenced by both their previous life experiences and their university education to date. Allied health students in this study demonstrated moderate HL assessed through quantitative data; however, they reported high HL when assessed qualitatively. Of interest is that the students in this study voiced that their HL was dynamic and multi-faceted. The authors propose that the qualitative measure of HL self-efficacy aligns with the definition of HL as the ability to access, understand, appraise, and use information” [1] given that students acknowledged that their base-level HL enabled them to continue to develop their HL. The authors propose that the recognition of a continually evolving HL and the self-efficacy to enact that is what enables increased quantitative scores on measures such as the HLQ. In respect to their roles as health professionals, allied health students anticipated that supporting clients’ HL was an innate and valuable aspect of their future roles as healthcare professionals, as they viewed HL as an integral aspect of client advocacy and person-centred care. The students’ perceptions of HL as patient advocacy and person-centred care as informed by their own experiences supports the inclusion of explicit HL curricula in healthcare professional education. Furthermore, it encourages educators to introduce HL as a framework for person-centred care early in healthcare education to encourage the development of person-centred capabilities amongst allied health students.

Allied health students in this study reflected positively on their own HL when they considered both their previous health history and their health in the future. Students attributed their level of HL to their previous life experiences and acknowledged the contribution of their university training prior to commencing their post-graduate studies. The students’ beliefs that their previous university education contributed to their HL supports similar research which reports that HL is developed following explicit and intentional training [12,13,15]. However, similarly to Sukys et al. [13], this research cannot draw conclusions regarding the causality of the association between university health education and positive associations with HL. Whilst the students’ previous healthcare-specific university study may have increased their HL, the pre-standing HL of the students influenced by their family environment may have led them to become healthcare professionals. The participants in this study had not received any specific HL content in their allied health programs prior to their completion of the survey; however, other factors not identified in the qualitative data may have contributed to their understanding of HL. The causal relationship between HL and university education presents as an area of research for stakeholders who aim to increase equity in university recruitment and retention amongst traditionally marginalised groups.

In addition to reporting perceived high levels of HL in qualitative data, the students in this study demonstrated similar HL to physiotherapists practicing in private practice [15]. Allied health students’ confidence in the knowledge domain of the HLQ was rated as 26.17 from a maximum possible score of 50. Practicing physiotherapists (*n* = 19) have previously rated their confidence in the knowledge domain of the HLQ prior to an HL training workshop as 26 [15]. Similarly, the allied health students’ confidence in the skills domain of the HLQ was rated as 14.87 from a maximum possible score of 25, compared to a score of 14.16 amongst the practicing physiotherapists [15]. Despite a small sample size of both cohorts, the similar confidence ratings in both knowledge and skills of Australian allied health students in this study and physiotherapists practising in Australia in Bird et al. (2022) suggests that HL must be explicitly addressed in health professional education, beyond the HL that is obtained incidentally through health-related study, to maximise the HL of graduating cohorts. The students in this study have demonstrated the values that underpin HL support for clients; however, further research is required to explore the application of this foundational theory to future skills and behaviours.

When reflecting on their future roles, allied health students perceived that being responsive to the HL of their clients and increasing the HL of their clients were vital components of their future roles and contributed to understanding allied health as altruistic professions. This perception was informed by their previous firsthand experiences of accessing healthcare. Within nursing literature, students’ previous healthcare exposure is understood to be the most influential factor when choosing nursing as a career [22]. When discussing their future roles, students in this study perceived that their desire for an altruistic career had led them to study allied health. Wu et al. (2015) undertook a systematic review of actors influencing career choice among healthcare students. The review found that the desire to help others, i.e., being altruistic, was a top factor influencing career choices amongst students in pharmacy, nursing, and medicine [23]. Encouragingly, students in this study voiced that their future roles in supporting clients’ HL through health promotion were part of their commitment to person-centred care and to encouraging client agency. This perception of encouraging client agency and person-centred care as true altruism is in line with current best practices supported by the Australian Commission of Safety and Quality in Healthcare [24].

Interestingly, students in this study voiced that appropriate communication for each clients’ unique level of HL was vital for supporting client’s HL, and equally important was communication within the inter-professional team to ensure unanimous health messaging. The importance of cooperation and communication within the interprofessional team commonly forms the basis of interprofessional frameworks, including the Canadian Interprofessional Health Collaborative (CIHC) Framework [25]. The goal of the CIHC Framework is interprofessional collaboration which is defined as “*a partnership between a team of health providers and a client in a participatory, collaborative, and coordinated approach to shared decision-making around health and social issues*” [25]. Positioning supporting a client’s HL within the CIHC Framework encourages healthcare providers to reflect on domains such as interprofessional communication and person-centred care, which are concepts relevant to HL that were discussed by the students in this study. Whilst it is not possible to isolate the students’ previous experiences with the CIHC Framework that may have informed their responses, the finding of this study supports research and teaching that consider HL using such frameworks. The use of an interprofessional framework such as the CIHC Framework may assist in the implementation of HL practices into pre-professional preparation for health promotion and the practice of health promotion with clients.

Allied health students in this study emphasised the importance of all healthcare professionals contributing to the ongoing support of clients’ HL; however, they did not demonstrate an understanding of the complex, dynamic, and context-specific nature of HL. During the interviews, students reflected on the dynamic and context-specific nature of their own HL; however, they did not discuss their clients’ HL as being similarly complex or context-specific. This finding provides direction for university educators for the scaffolding of HL skills across curricula. An adult learning theory such as the transformative learning theory of critical reflection by Mezirow would be one appropriate approach for the development of HL competency within post-graduate allied health curricula [26]. The transformative learning theory of critical reflection encourages learners to challenge their embedded assumptions as their frame of reference evolves [26,27]. In post-graduate and undergraduate pharmacy students, transformative and critically reflective teaching strategies are known to allow students to provide more tailored care to their clients [28]. This may also apply when tailoring allied health care to be at an appropriate level of HL for individual clients. Interestingly, a transformative learning model of critical reflection may be a useful framework for both pre-professional preparation for HL amongst allied health students and for supporting clients to improve their HL given that the framework respects prior knowledge and learning [26]. Ultimately, stakeholders in health professional education are encouraged to acknowledge the established HL of students prior to the commencement of post-graduate studies to ensure that HL curricula are designed at the appropriate level.

Encouragingly, students in this study acknowledged the value of opportunities to learn about HL early in their course, as it allowed them to apply an HL lens to their concurrent learning and, hypothetically, their future learning. Despite the need to explicitly address HL in health professional curricula [9], HL has been identified as a gap in current curricula [15,29]. The need to address HL during pre-professional curricula is further supported by new-graduate experiences. In the United Kingdom, Chesterton et al. (2021) explored new-graduate physiotherapists perceived preparedness for clinical practice and concluded that greater emphasis is required on content that contributes to the “*production of increased health literacy, linked directly to healthcare service utilisation and ultimately patient outcomes*” (page 7) [29]. Similarly, an Australian Systematic Review of HL training in health professions education advocates for increasing opportunities for HL inter-professional education, given the findings of the review that HL is an underdeveloped domain in the health professional education field [9]. Stand-alone approaches to pre-professional HL training have been published, for example, by Doyle et al. (2013), who conducted an HL assignment amongst first-year medical and physiotherapy students. In their evaluation, Doyle et al. (2013) noted the value of introducing HL early in students’ educational journeys to enable students to build HL into their clinical competencies throughout the remainder of their curriculum [30]. The findings of this study support prioritising HL early in a curriculum so that students are equipped with an HL lens throughout the course of their learning and can subsequently apply these skills during opportunities for health promotion with clients. Further, we see value in tracking how HL-related knowledge, skills, and professional attributes develop over time and alongside other areas, such as interprofessional collaborative practice capabilities, providing direction for future research.

### Limitations

There are limitations of this study that must be acknowledged. Firstly, the sample was restricted to allied health students from one university, which limits the generalisability of the findings. Secondly, there may be a selection bias present as students who held particularly positive or negative perceptions of HL may have been more likely to consent to participation in the study. Additionally, there may be a response bias as students were aware of the content of the study and may have provided responses that they perceived to be valuable to the researchers. The limitation of the use of telephone interviews must also be considered, given their restriction on non-verbal communication and rapport building. Finally, the sample size required to ensure appropriate power of the HLQ in cross-sectional studies is not known. The modest sample size of the survey participants and the interview participants in this study may also have impacted the results.

## 5. Conclusions

This study provides data on both the HL of allied health students and their perceptions of their future roles in HL. Allied health students’ confidence in the knowledge domain of the HLQ was rated as 26.17 from a maximum possible score of 50, and their confidence in the skills domain of the HLQ was rated as 14.87 from a maximum possible score of 25. Allied health students perceived that being responsive to HL and supporting clients to improve their HL are both significant and valuable aspects of their future roles as health professionals. Allied health students in this study identified that their previous life experiences and their prior health-related education both contributed to their HL. The authors propose that the recognition of a continually evolving HL and the self-efficacy to enact that is what enables increased quantitative scores on measures such as the HLQ. University educators are encouraged to utilise students’ strong perceptions of their role in supporting clients’ HL early in pre-professional preparation to improve the HL capabilities of the new graduates entering the workforce.

## Figures and Tables

**Table 2:** Characteristics of survey participants.

Demographic Characteristics	Participants (*n* = Frequency) (%)
Gender	
Male	12/32 (37.50%)
Female	20/32 (62.50%)
Program of study	
Master of Speech Pathology	10/32 (31.25%)
Master of Physiotherapy	22/32 (68.75%)

**Table 3:** Participants (*n* = 30) HLQ results.

On a Scale from 1 to 5 (1 Is Lowest, Not Confident at All; 5 Is Highest, Very Confident), Please Indicate Your Level of Confidence in Being Able to Do the Following:	
Item:	Mean (SD)
1. Explain differences in the various ways that health literacy is defined and conceptualised.	2.87 (0.57)
2. Outline client/patient indicators and outcomes of low health literacy.	3.03 (0.81)
3. Explain that it is the responsibility of the health and human services sector to address the is match between client/patient and service provider communication.	2.87 (0.73)
4. Explain the relationship between health literacy and health equity.	3.00 (0.79)
5. Identify population groups that are at increased risk of low health literacy.	3.60 (0.62)
6. Discuss the relationship between health literacy, cultural competence, and consumer participation.	2.70 (0.84)
7. Outline the risk management and quality improvement imperatives of improved health literacy.	2.57 (0.90)
8. Describe the rationale for applying a universal precaution approach to health literacy.	2.70 (0.84)
9. Recognise, avoid and/or constructively correct the use of professional jargon.	3.33 (0.80)
10. Effectively use the Teach-Back technique to assess client/patients’ understanding of health and wellbeing information.	2.67 (1.18)
11. Use plain language principles in written communication.	3.50 (0.68)
12. Integrate health literacy into risk management and quality improvement strategies.	2.93 (0.98)
13. Outline the attributes of a health literate organisation.	2.40 (0.72)
14. Develop a strategy that will prepare the workforce within your organisation to be health literate.	2.43 (0.94)
15. Explain the rationale for including representatives of your client group in the design, implementation, and evaluation of health and wellbeing information and services.	2.83 (0.83)

**Table 4:** Participants’ (*n* = 30) health literacy knowledge and skills from the HLQ domains.

	Mean
Knowledge *	28.57
Skills **	14.87

* Knowledge is based on the sum of questions 1–8, 13, and 15 of the HLQ; ** Skills is based on the sum of questions 9–12 and 14 of the HLQ.

**Table 5:** Interview Participant Demographic Details.

#	Gender	Age	Program of Study	Previous Degree of Study
1	Female	25	Speech Pathology	Bachelor of Arts
2	Female	26	Speech Pathology	Bachelor of Psychological Sciences
3	Male	22	Physiotherapy	Bachelor of Medical Research
4	Female	33	Physiotherapy	Bachelor of Exercise Science
5	Male	52	Physiotherapy	Bachelor of Exercise and Sport Science
6	Male	23	Physiotherapy	Bachelor of Exercise and Sport Science

## Data Availability

The data presented in this study are available on request from the corresponding author. The data are not publicly available due to privacy concerns.
