# Correction: Martin et al. The Health Literacy of First Year Physiotherapy and Speech Pathology Students and Their Perceived Future Roles in Supporting Their Clients’ Health Literacy. *Int. J. Environ. Res. Public Health* 2023, *20*, 6013

**DOI:** 10.3390/ijerph22071134

**Published:** 2025-07-18

**Authors:** Romany Martin, Jade Cartwright, Marie-Louise Bird

**Affiliations:** School of Health Sciences, University of Tasmania, Launceston, TAS 7250, Australia

The authors have requested that the following changes be made to the original publication [1]: Osborne et al. (2013) [2] was incorrectly cited as the source of the health literacy questionnaire that was used to collect quantitative data. Instead, the questionnaire that was used to collect the data in the manuscript is the health literacy questionnaire by the Centre for Culture, Ethnicity, and Health (2016) [3,4]. The citation has now been corrected, and minor changes have been made throughout the manuscript to reflect this correction.

Section 2, paragraph 1 should read

A convergent parallel mixed-methods design was undertaken to achieve the aims of the study, with pragmatism as an umbrella philosophy [16]. A health literacy questionnaire (HLQ) [15] which has been previously used to assess HL knowledge and skill amongst practicing physiotherapists was administered via hard-copy survey. The survey was originally created by the Centre for Culture, Ethnicity, and Health [17].

Section 2.2, paragraph 1 should read

The HLQ chosen has featured in the literature and has been used to identify the HL skills and professional development needs of physiotherapists [15]. Whilst the HLQ used in this study aims to measure the HL of survey respondents similar to other HLQ tools [18], it may also measure elements of health literacy responsiveness.

The following two references have been removed:

17. Osborne, R.H.; Batterham, R.W.; Elsworth, G.R.; Hawkins, M.; Buchbinder, R. The grounded psychometric development and initial validation of the Health Literacy Questionnaire (HLQ). *BMC Public Health* **2013**, *13*, 658. https://doi.org/10.1186/1471-2458-13-658.
18. Hawkins, M.; Gill, S.D.; Batterham, R.; Elsworth, G.R.; Osborne, R.H. The Health Literacy Questionnaire (HLQ) at the patient-clinician interface: A qualitative study of what patients and clinicians mean by their HLQ scores. *BMC Health Serv. Res.* **2017**, *17*, 309.

The following two references have been added:

17. Centre for Culture, Ethnicity, and Health. Health Literacy Background Paper. 2016. Available online: https://www.ceh.org.au/ (accessed on 26 July 2022).
18. Osborne, R.H.; Batterham, R.W.; Elsworth, G.R.; Hawkins, M.; Buchbinder, R. The grounded psychometric development and initial validation of the Health Literacy Questionnaire (HLQ). *BMC Public Health* **2013**, *13*, 658. https://doi.org/10.1186/1471-2458-13-658.

In Abstract, the sentence “Data collected included the Health Literacy Questionnaire (HLQ) (*n* = 30) and qualitative telephone interviews (*n* = 6).” was replaced with “Data collected included a Health Literacy Questionnaire (HLQ) (*n* = 30) and qualitative telephone interviews (*n* = 6).”.

In 1. Introduction, on paragraph 4, the sentence “This has important implications for tailoring health professional curricula in the pursuit of HL-based competence, as students enrolled in the post-graduate entry-level master’s programs have higher Health Literacy Questionnaire (HLQ) scores than those enrolled in bachelor’s programs [12].” was replaced with “This has important implications for tailoring health professional curricula in the pursuit of HL-based competence, as students enrolled in the post-graduate entry-level master’s programs have higher HL scores than those enrolled in bachelor’s programs [12].”.

In Limitations, the sentence “Finally, whilst the HLQ has been established to be both valid and reliable, the sample size required to ensure appropriate power of the HLQ in cross-sectional studies is not known.” was replaced with “Finally, the sample size required to ensure appropriate power of the HLQ in cross-sectional studies is not known.”.

With this correction, the order of some references has been adjusted accordingly. The authors apologize for any inconvenience. The authors state that the scientific conclusions are unaffected. This correction was approved by the Academic Editor. The original publication has also been updated.
